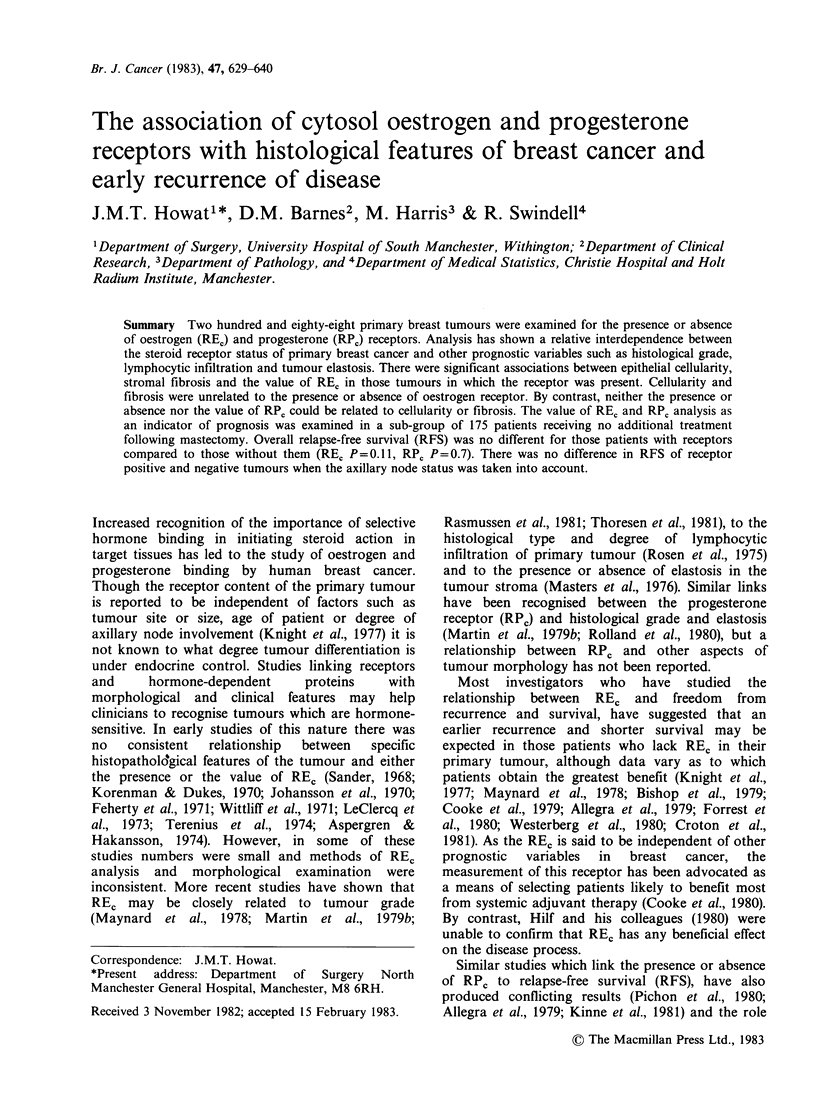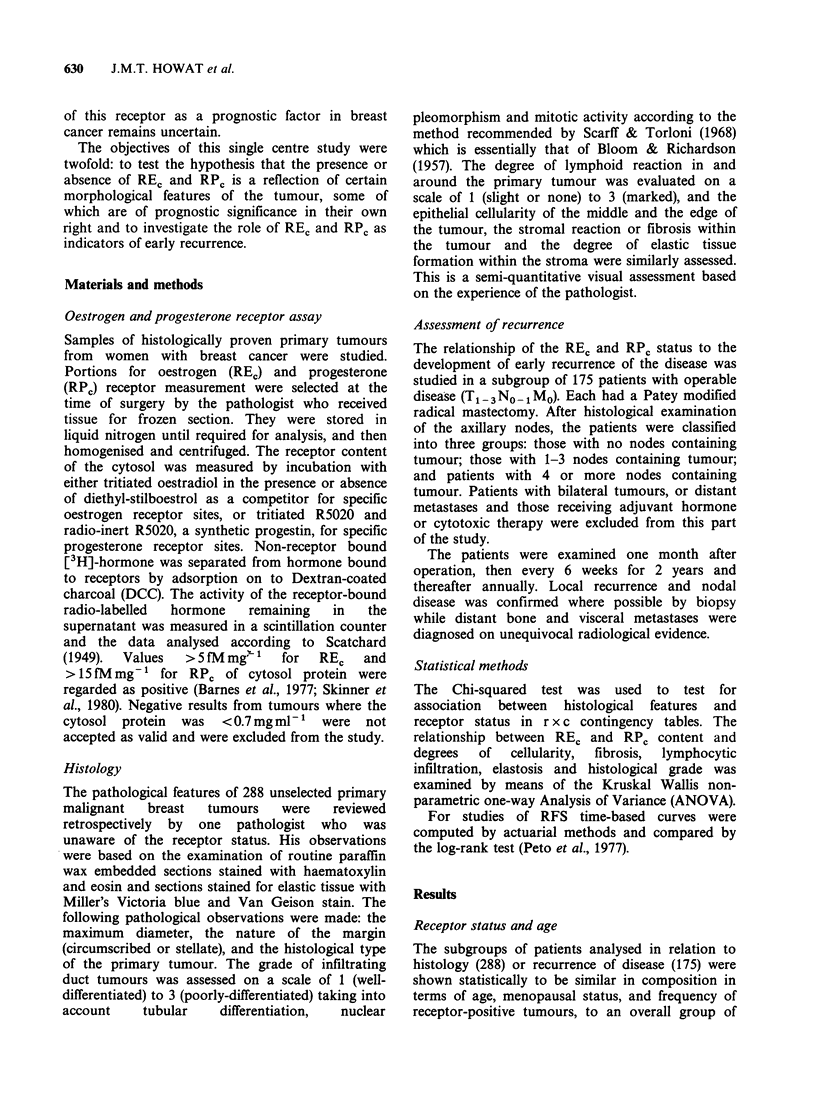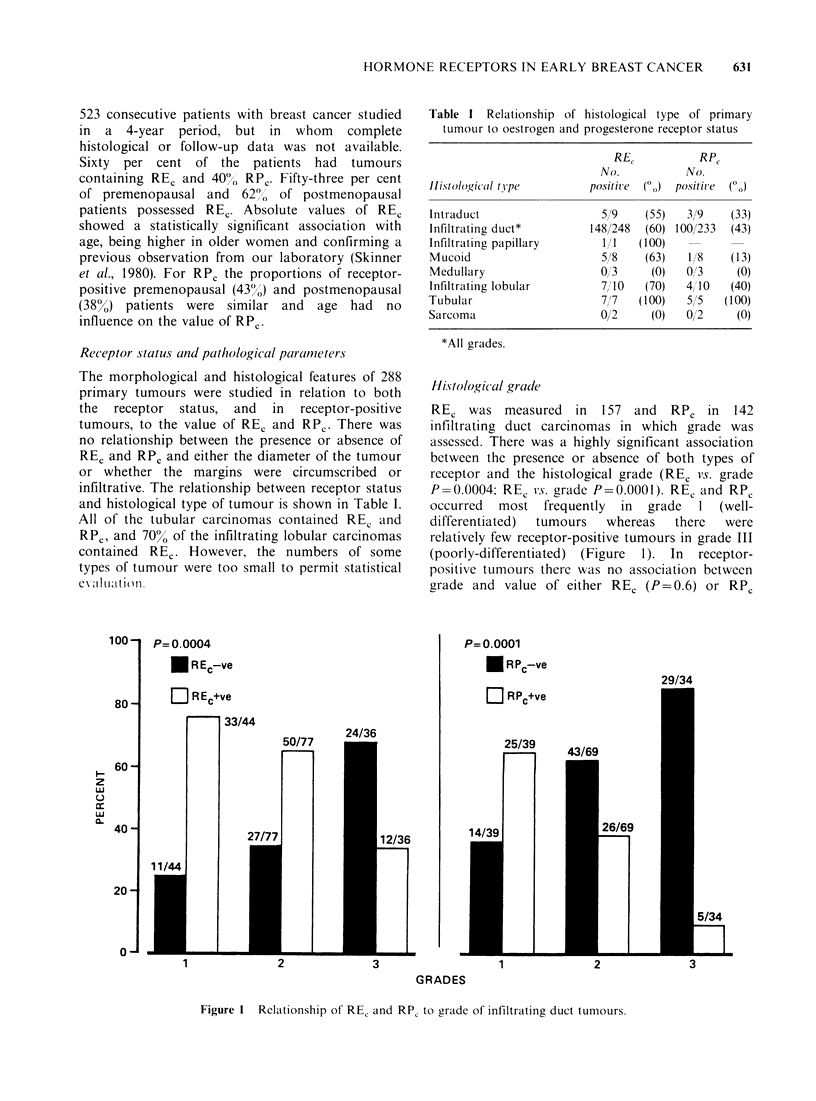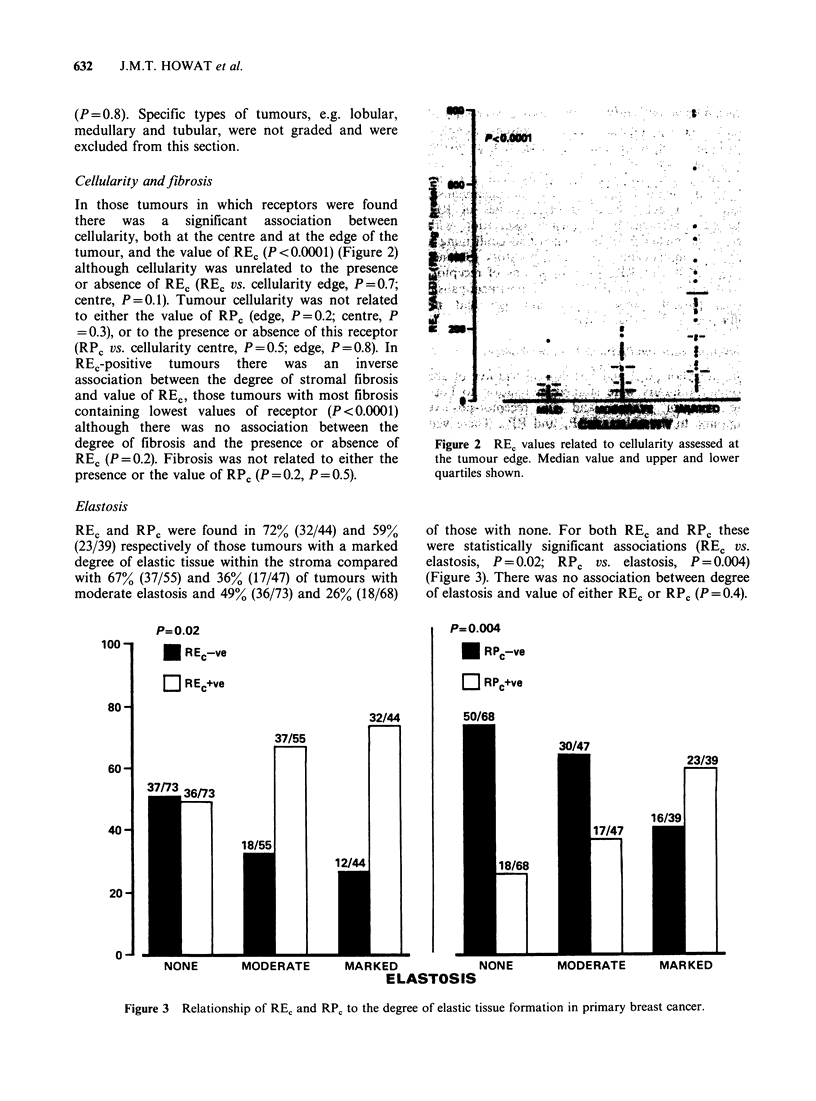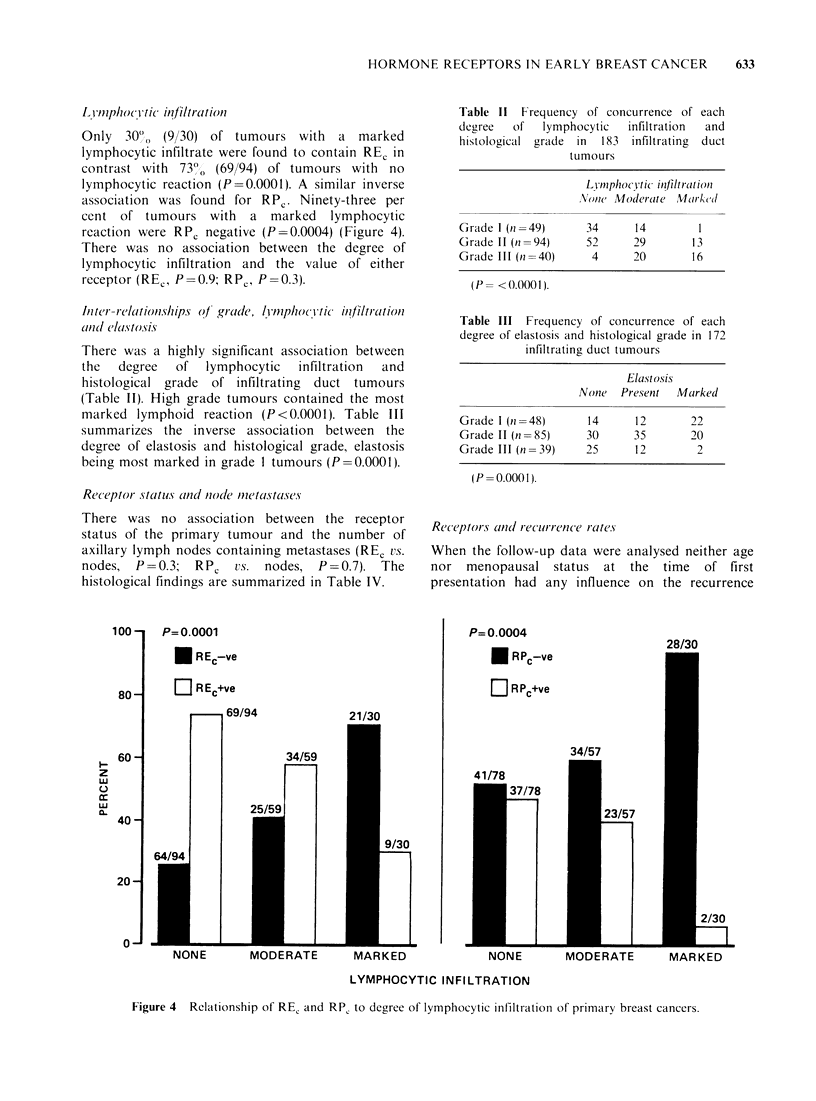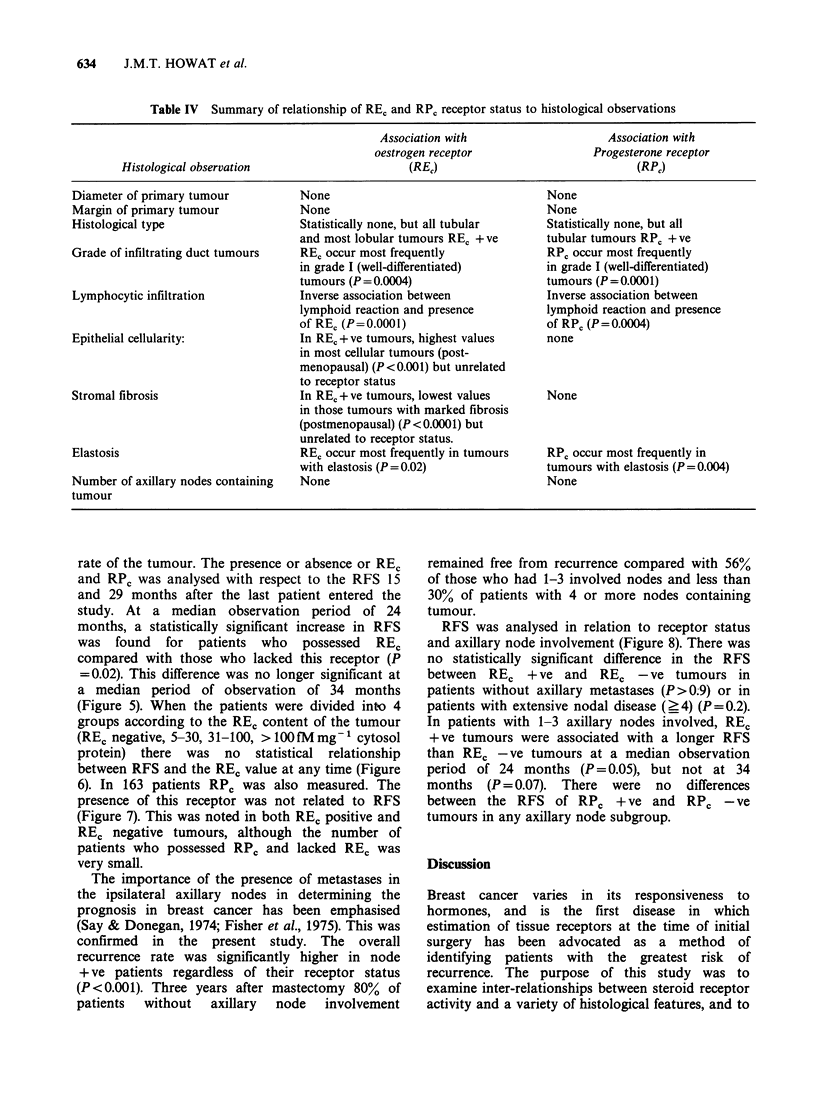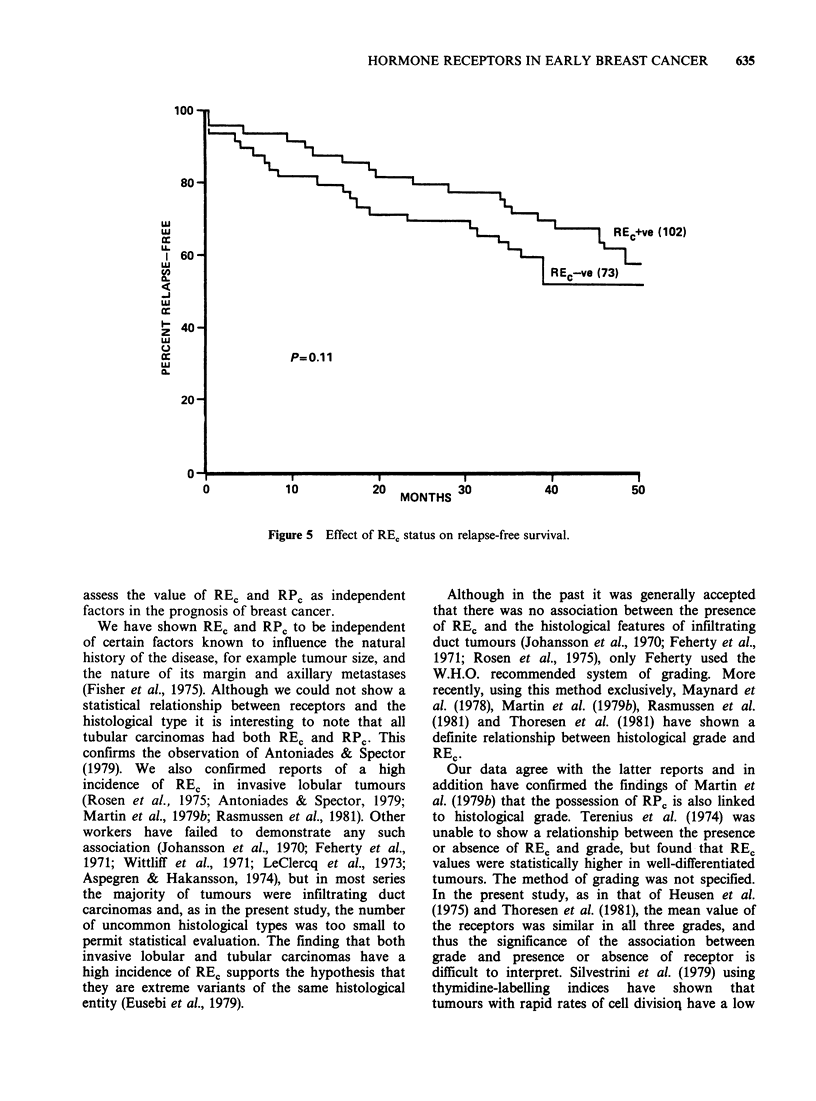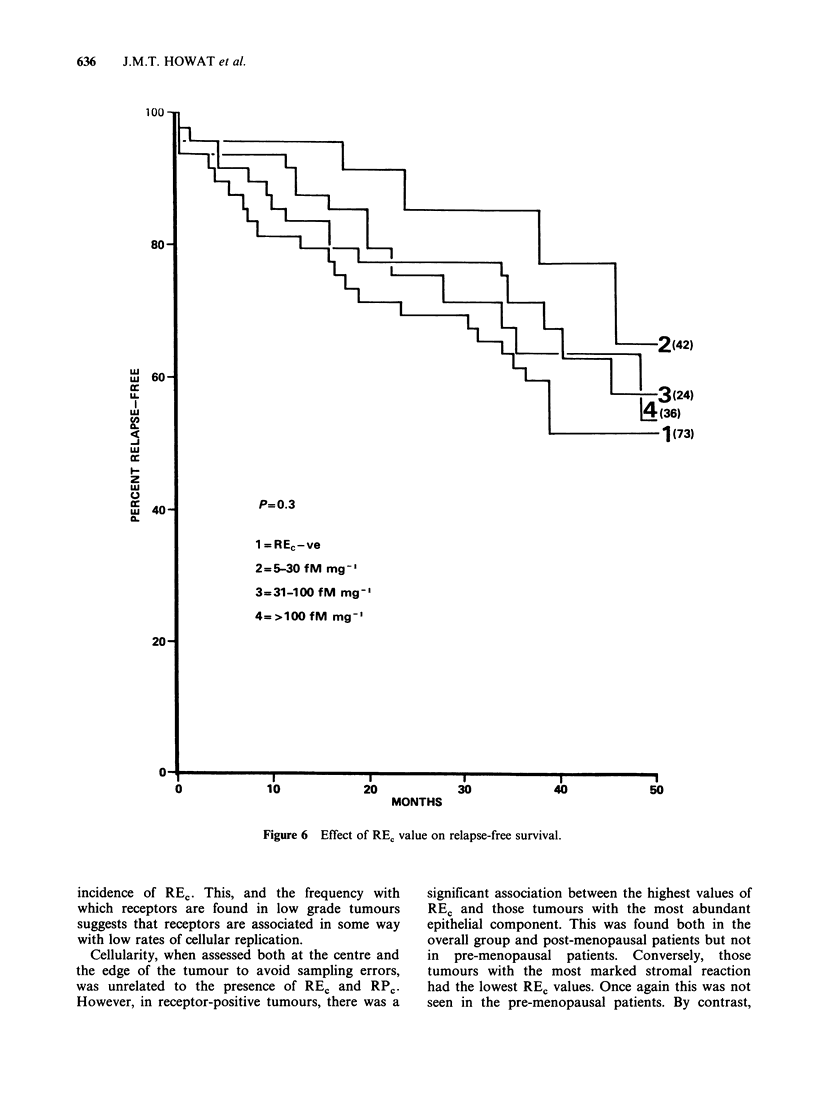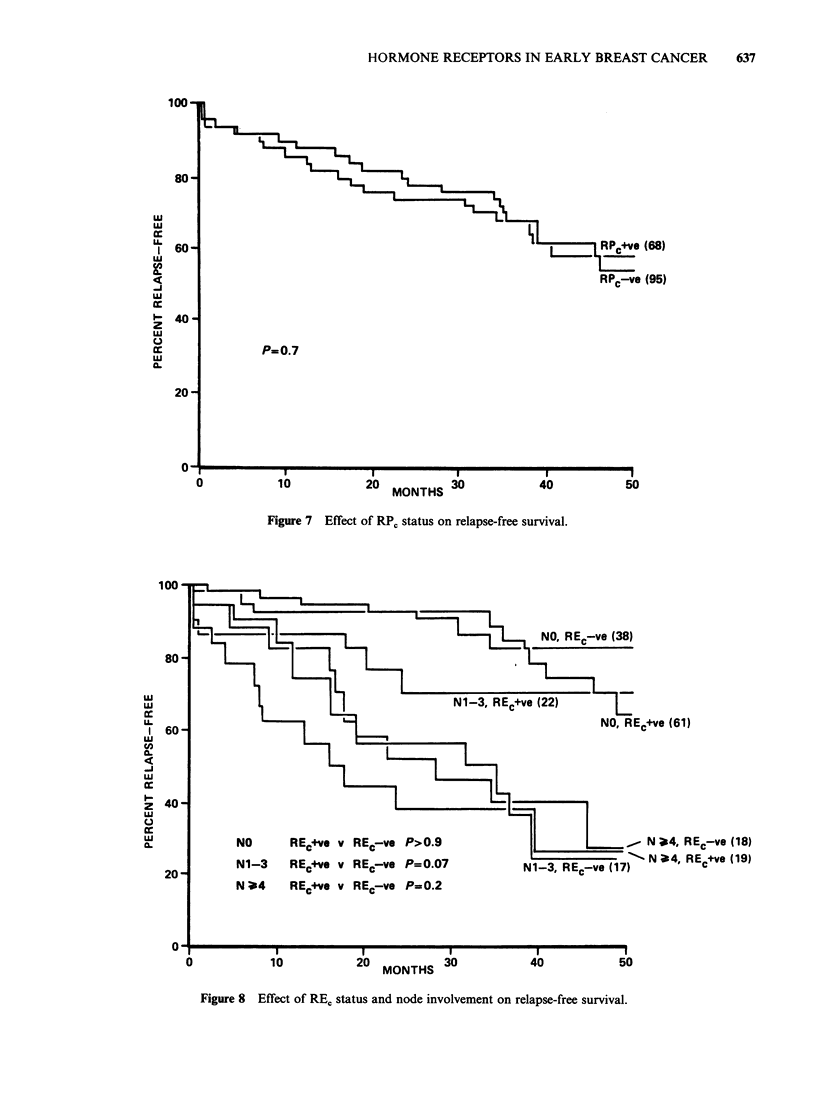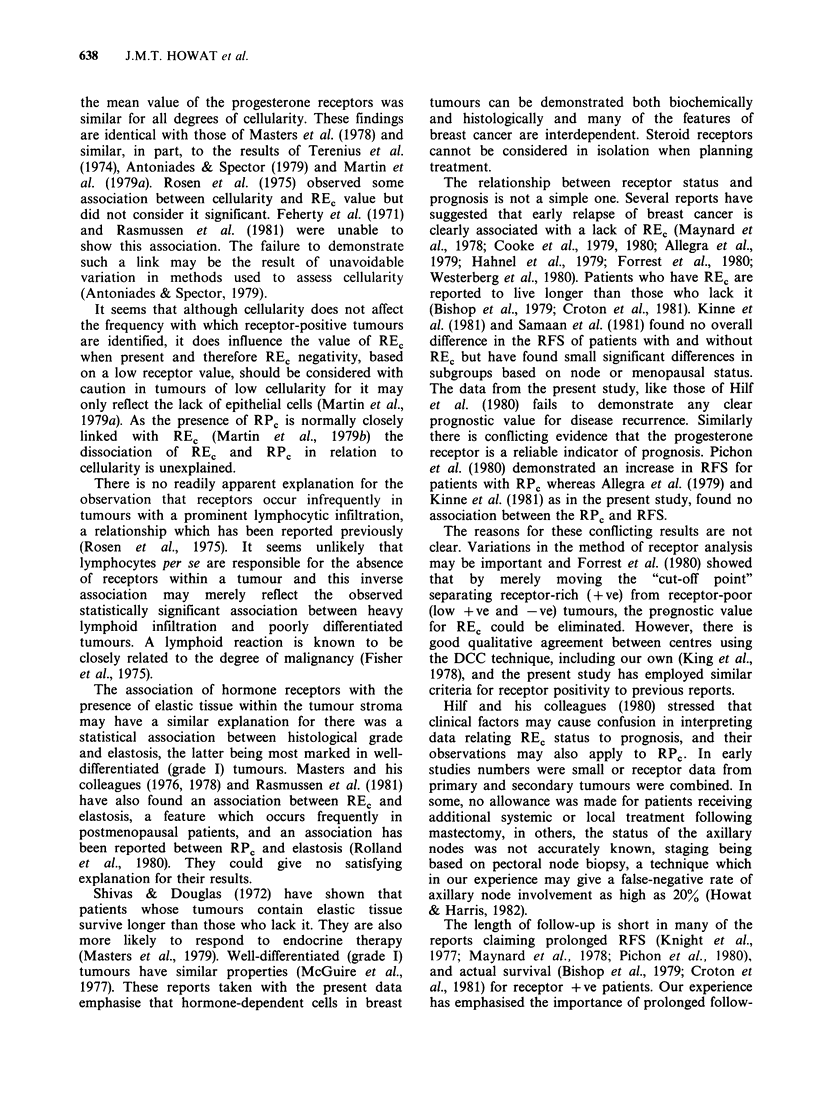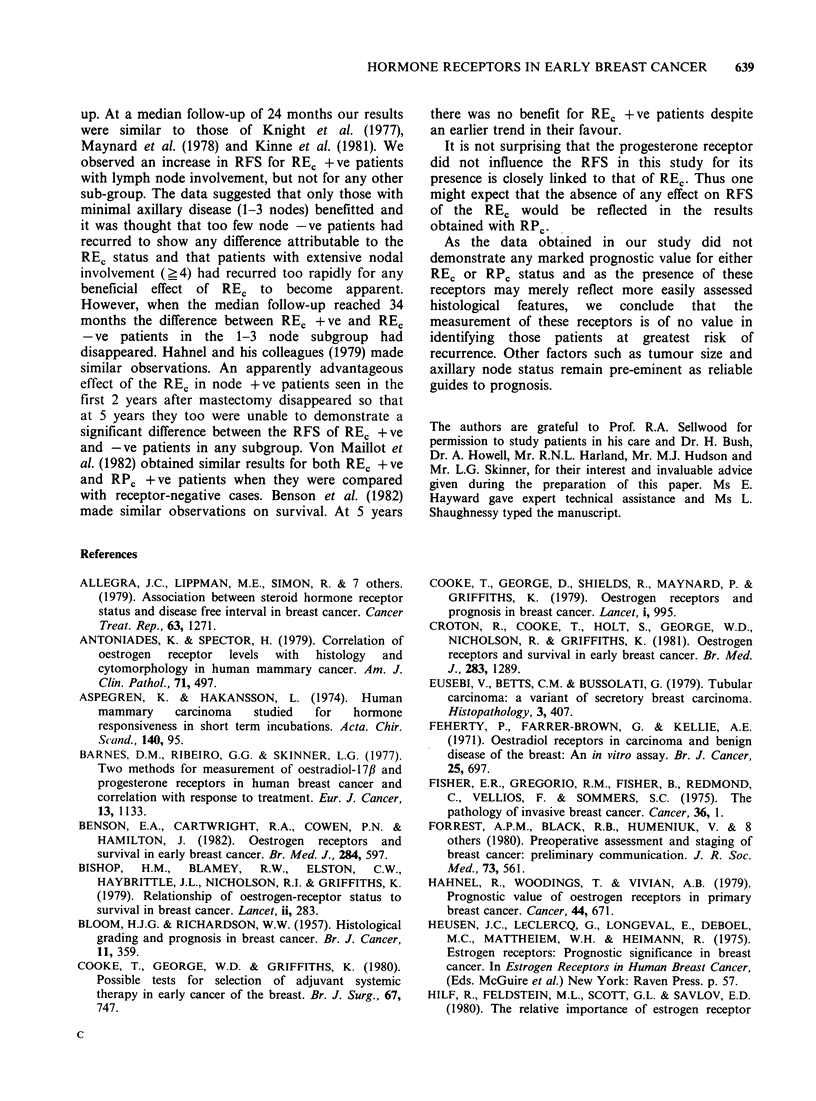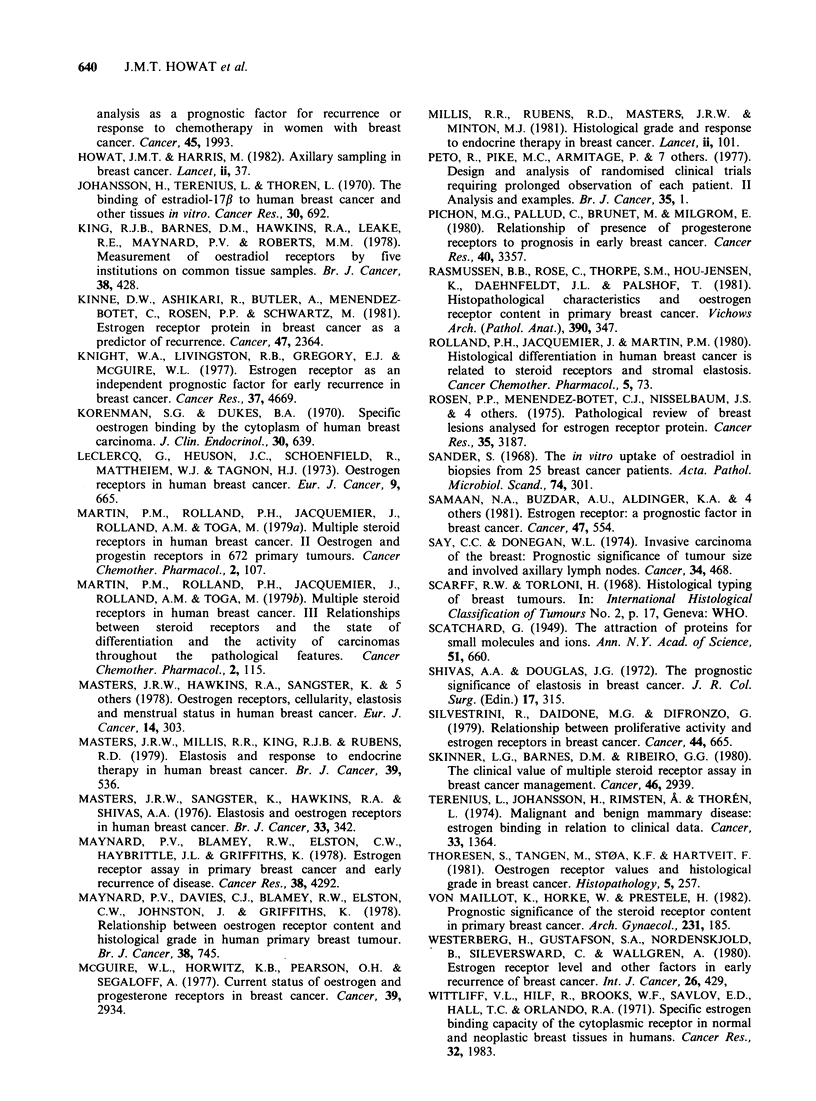# The association of cytosol oestrogen and progesterone receptors with histological features of breast cancer and early recurrence of disease.

**DOI:** 10.1038/bjc.1983.101

**Published:** 1983-05

**Authors:** J. M. Howat, D. M. Barnes, M. Harris, R. Swindell

## Abstract

Two hundred and eighty-eight primary breast tumours were examined for the presence or absence of oestrogen (REc) and progesterone (RPc) receptors. Analysis has shown a relative interdependence between the steroid receptor status of primary breast cancer and other prognostic variables such as histological grade, lymphocytic infiltration and tumour elastosis. There were significant associations between epithelial cellularity, stromal fibrosis and the value of REc in those tumours in which the receptor was present. Cellularity and fibrosis were unrelated to the presence or absence of oestrogen receptor. By contrast, neither the presence or absence nor the value of RPc could be related to cellularity or fibrosis. The value of REc and RPc analysis as an indicator of prognosis was examined in a sub-group of 175 patients receiving no additional treatment following mastectomy. Overall relapse-free survival (RFS) was no different for those patients with receptors compared to those without them (REc P = 0.11, RPc P = 0.7). There was no difference in RFS of receptor positive and negative tumours when the axillary node status was taken into account.


Br. J. Cancer (1983), **47**, 629–640

### The association of cytosol oestrogen and progesterone receptors with histological features of breast cancer and early recurrence of disease

J.M.T. Howat[1]*, D.M. Barnes[2], M. Harris[3] & R. Swindell[4]

[1]*Department of Surgery, University Hospital of South Manchester, Withington;* [2]*Department of Clinical Research,* [3]*Department of Pathology, and* [4]*Department of Medical Statistics, Christie Hospital and Holt Radium Institute, Manchester.*

**Summary** Two hundred and eighty-eight primary breast tumours were examined for the presence or absence of oestrogen ($RE_c$) and progesterone ($RP_c$) receptors. Analysis has shown a relative interdependence between the steroid receptor status of primary breast cancer and other prognostic variables such as histological grade, lymphocytic infiltration and tumour elastosis. There were significant associations between epithelial cellularity, stromal fibrosis and the value of $RE_c$ in those tumours in which the receptor was present. Cellularity and fibrosis were unrelated to the presence or absence of oestrogen receptor. By contrast, neither the presence or absence nor the value of $RP_c$ could be related to cellularity or fibrosis. The value of $RE_c$ and $RP_c$ analysis as an indicator of prognosis was examined in a sub-group of 175 patients receiving no additional treatment following mastectomy. Overall relapse-free survival (RFS) was no different for those patients with receptors compared to those without them ($RE_c$ $P=0.11$, $RP_c$ $P=0.7$). There was no difference in RFS of receptor positive and negative tumours when the axillary node status was taken into account.

Increased recognition of the importance of selective hormone binding in initiating steroid action in target tissues has led to the study of oestrogen and progesterone binding by human breast cancer. Though the receptor content of the primary tumour is reported to be independent of factors such as tumour site or size, age of patient or degree of axillary node involvement (Knight *et al.*, 1977) it is not known to what degree tumour differentiation is under endocrine control. Studies linking receptors and hormone-dependent proteins with morphological and clinical features may help clinicians to recognise tumours which are hormone-sensitive. In early studies of this nature there was no consistent relationship between specific histopathological features of the tumour and either the presence or the value of $RE_c$ (Sander, 1968; Korenman & Dukes, 1970; Johansson *et al.*, 1970; Feherty *et al.*, 1971; Wittliff *et al.*, 1971; LeClercq *et al.*, 1973; Terenius *et al.*, 1974; Aspergren & Hakansson, 1974). However, in some of these studies numbers were small and methods of $RE_c$ analysis and morphological examination were inconsistent. More recent studies have shown that $RE_c$ may be closely related to tumour grade (Maynard *et al.*, 1978; Martin *et al.*, 1979*b*; Rasmussen *et al.*, 1981; Thoresen *et al.*, 1981), to the histological type and degree of lymphocytic infiltration of primary tumour (Rosen *et al.*, 1975) and to the presence or absence of elastosis in the tumour stroma (Masters *et al.*, 1976). Similar links have been recognised between the progesterone receptor ($RP_c$) and histological grade and elastosis (Martin *et al.*, 1979*b*; Rolland *et al.*, 1980), but a relationship between $RP_c$ and other aspects of tumour morphology has not been reported.

Most investigators who have studied the relationship between $RE_c$ and freedom from recurrence and survival, have suggested that an earlier recurrence and shorter survival may be expected in those patients who lack $RE_c$ in their primary tumour, although data vary as to which patients obtain the greatest benefit (Knight *et al.*, 1977; Maynard *et al.*, 1978; Bishop *et al.*, 1979; Cooke *et al.*, 1979; Allegra *et al.*, 1979; Forrest *et al.*, 1980; Westerberg *et al.*, 1980; Croton *et al.*, 1981). As the $RE_c$ is said to be independent of other prognostic variables in breast cancer, the measurement of this receptor has been advocated as a means of selecting patients likely to benefit most from systemic adjuvant therapy (Cooke *et al.*, 1980). By contrast, Hilf and his colleagues (1980) were unable to confirm that $RE_c$ has any beneficial effect on the disease process.

Similar studies which link the presence or absence of $RP_c$ to relapse-free survival (RFS), have also produced conflicting results (Pichon *et al.*, 1980; Allegra *et al.*, 1979; Kinne *et al.*, 1981) and the role

Correspondence: J.M.T. Howat.
*Present address: Department of Surgery North Manchester General Hospital, Manchester, M8 6RH.

Received 3 November 1982; accepted 15 February 1983.

© The Macmillan Press Ltd., 1983

of this receptor as a prognostic factor in breast cancer remains uncertain.

The objectives of this single centre study were twofold: to test the hypothesis that the presence or absence of $RE_c$ and $RP_c$ is a reflection of certain morphological features of the tumour, some of which are of prognostic significance in their own right and to investigate the role of $RE_c$ and $RP_c$ as indicators of early recurrence.

### Materials and methods

*Oestrogen and progesterone receptor assay*

Samples of histologically proven primary tumours from women with breast cancer were studied. Portions for oestrogen ($RE_c$) and progesterone ($RP_c$) receptor measurement were selected at the time of surgery by the pathologist who received tissue for frozen section. They were stored in liquid nitrogen until required for analysis, and then homogenised and centrifuged. The receptor content of the cytosol was measured by incubation with either tritiated oestradiol in the presence or absence of diethyl-stilboestrol as a competitor for specific oestrogen receptor sites, or tritiated R5020 and radio-inert R5020, a synthetic progestin, for specific progesterone receptor sites. Non-receptor bound $[^3H]$-hormone was separated from hormone bound to receptors by adsorption on to Dextran-coated charcoal (DCC). The activity of the receptor-bound radio-labelled hormone remaining in the supernatant was measured in a scintillation counter and the data analysed according to Scatchard (1949). Values $>5\,fM\,mg^{-1}$ for $RE_c$ and $>15\,fM\,mg^{-1}$ for $RP_c$ of cytosol protein were regarded as positive (Barnes *et al.*, 1977; Skinner *et al.*, 1980). Negative results from tumours where the cytosol protein was $<0.7\,mg\,ml^{-1}$ were not accepted as valid and were excluded from the study.

*Histology*

The pathological features of 288 unselected primary malignant breast tumours were reviewed retrospectively by one pathologist who was unaware of the receptor status. His observations were based on the examination of routine paraffin wax embedded sections stained with haematoxylin and eosin and sections stained for elastic tissue with Miller's Victoria blue and Van Geison stain. The following pathological observations were made: the maximum diameter, the nature of the margin (circumscribed or stellate), and the histological type of the primary tumour. The grade of infiltrating duct tumours was assessed on a scale of 1 (well-differentiated) to 3 (poorly-differentiated) taking into account tubular differentiation, nuclear pleomorphism and mitotic activity according to the method recommended by Scarff & Torloni (1968) which is essentially that of Bloom & Richardson (1957). The degree of lymphoid reaction in and around the primary tumour was evaluated on a scale of 1 (slight or none) to 3 (marked), and the epithelial cellularity of the middle and the edge of the tumour, the stromal reaction or fibrosis within the tumour and the degree of elastic tissue formation within the stroma were similarly assessed. This is a semi-quantitative visual assessment based on the experience of the pathologist.

*Assessment of recurrence*

The relationship of the $RE_c$ and $RP_c$ status to the development of early recurrence of the disease was studied in a subgroup of 175 patients with operable disease ($T_{1-3}N_{0-1}M_0$). Each had a Patey modified radical mastectomy. After histological examination of the axillary nodes, the patients were classified into three groups: those with no nodes containing tumour; those with 1–3 nodes containing tumour; and patients with 4 or more nodes containing tumour. Patients with bilateral tumours, or distant metastases and those receiving adjuvant hormone or cytotoxic therapy were excluded from this part of the study.

The patients were examined one month after operation, then every 6 weeks for 2 years and thereafter annually. Local recurrence and nodal disease was confirmed where possible by biopsy while distant bone and visceral metastases were diagnosed on unequivocal radiological evidence.

*Statistical methods*

The Chi-squared test was used to test for association between histological features and receptor status in $r \times c$ contingency tables. The relationship between $RE_c$ and $RP_c$ content and degrees of cellularity, fibrosis, lymphocytic infiltration, elastosis and histological grade was examined by means of the Kruskal Wallis non-parametric one-way Analysis of Variance (ANOVA).

For studies of RFS time-based curves were computed by actuarial methods and compared by the log-rank test (Peto *et al.*, 1977).

### Results

*Receptor status and age*

The subgroups of patients analysed in relation to histology (288) or recurrence of disease (175) were shown statistically to be similar in composition in terms of age, menopausal status, and frequency of receptor-positive tumours, to an overall group of

523 consecutive patients with breast cancer studied in a 4-year period, but in whom complete histological or follow-up data was not available. Sixty per cent of the patients had tumours containing $RE_c$ and 40% $RP_c$. Fifty-three per cent of premenopausal and 62% of postmenopausal patients possessed $RE_c$. Absolute values of $RE_c$ showed a statistically significant association with age, being higher in older women and confirming a previous observation from our laboratory (Skinner *et al.*, 1980). For $RP_c$ the proportions of receptor-positive premenopausal (43%) and postmenopausal (38%) patients were similar and age had no influence on the value of $RP_c$.

*Receptor status and pathological parameters*

The morphological and histological features of 288 primary tumours were studied in relation to both the receptor status, and in receptor-positive tumours, to the value of $RE_c$ and $RP_c$. There was no relationship between the presence or absence of $RE_c$ and $RP_c$ and either the diameter of the tumour or whether the margins were circumscribed or infiltrative. The relationship between receptor status and histological type of tumour is shown in Table I. All of the tubular carcinomas contained $RE_c$ and $RP_c$, and 70% of the infiltrating lobular carcinomas contained $RE_c$. However, the numbers of some types of tumour were too small to permit statistical evaluation.

**Table I** Relationship of histological type of primary tumour to oestrogen and progesterone receptor status

| *Histological type* | *$RE_c$ No. positive* | (%) | *$RP_c$ No. positive* | (%) |
|---|---|---|---|---|
| Intraduct | 5/9 | (55) | 3/9 | (33) |
| Infiltrating duct* | 148/248 | (60) | 100/233 | (43) |
| Infiltrating papillary | 1/1 | (100) | — | — |
| Mucoid | 5/8 | (63) | 1/8 | (13) |
| Medullary | 0/3 | (0) | 0/3 | (0) |
| Infiltrating lobular | 7/10 | (70) | 4/10 | (40) |
| Tubular | 7/7 | (100) | 5/5 | (100) |
| Sarcoma | 0/2 | (0) | 0/2 | (0) |

*All grades.

*Histological grade*

$RE_c$ was measured in 157 and $RP_c$ in 142 infiltrating duct carcinomas in which grade was assessed. There was a highly significant association between the presence or absence of both types of receptor and the histological grade ($RE_c$ *vs.* grade $P=0.0004$; $RE_c$ *vs.* grade $P=0.0001$). $RE_c$ and $RP_c$ occurred most frequently in grade I (well-differentiated) tumours whereas there were relatively few receptor-positive tumours in grade III (poorly-differentiated) (Figure 1). In receptor-positive tumours there was no association between grade and value of either $RE_c$ ($P=0.6$) or $RP_c$

**Figure 1** Relationship of $RE_c$ and $RP_c$ to grade of infiltrating duct tumours.

($P=0.8$). Specific types of tumours, e.g. lobular, medullary and tubular, were not graded and were excluded from this section.

*Cellularity and fibrosis*

In those tumours in which receptors were found there was a significant association between cellularity, both at the centre and at the edge of the tumour, and the value of $RE_c$ ($P<0.0001$) (Figure 2) although cellularity was unrelated to the presence or absence of $RE_c$ ($RE_c$ *vs.* cellularity edge, $P=0.7$; centre, $P=0.1$). Tumour cellularity was not related to either the value of $RP_c$ (edge, $P=0.2$; centre, $P=0.3$), or to the presence or absence of this receptor ($RP_c$ *vs.* cellularity centre, $P=0.5$; edge, $P=0.8$). In $RE_c$-positive tumours there was an inverse association between the degree of stromal fibrosis and value of $RE_c$, those tumours with most fibrosis containing lowest values of receptor ($P<0.0001$) although there was no association between the degree of fibrosis and the presence or absence of $RE_c$ ($P=0.2$). Fibrosis was not related to either the presence or the value of $RP_c$ ($P=0.2$, $P=0.5$).

**Figure 2** $RE_c$ values related to cellularity assessed at the tumour edge. Median value and upper and lower quartiles shown.

*Elastosis*

$RE_c$ and $RP_c$ were found in 72% (32/44) and 59% (23/39) respectively of those tumours with a marked degree of elastic tissue within the stroma compared with 67% (37/55) and 36% (17/47) of tumours with moderate elastosis and 49% (36/73) and 26% (18/68) of those with none. For both $RE_c$ and $RP_c$ these were statistically significant associations ($RE_c$ *vs.* elastosis, $P=0.02$; $RP_c$ *vs.* elastosis, $P=0.004$) (Figure 3). There was no association between degree of elastosis and value of either $RE_c$ or $RP_c$ ($P=0.4$).

**Figure 3** Relationship of $RE_c$ and $RP_c$ to the degree of elastic tissue formation in primary breast cancer.

*Lymphocytic infiltration*

Only 30% (9/30) of tumours with a marked lymphocytic infiltrate were found to contain $RE_c$ in contrast with 73% (69/94) of tumours with no lymphocytic reaction ($P=0.0001$). A similar inverse association was found for $RP_c$. Ninety-three per cent of tumours with a marked lymphocytic reaction were $RP_c$ negative ($P=0.0004$) (Figure 4). There was no association between the degree of lymphocytic infiltration and the value of either receptor ($RE_c$, $P=0.9$; $RP_c$, $P=0.3$).

*Inter-relationships of grade, lymphocytic infiltration and elastosis*

There was a highly significant association between the degree of lymphocytic infiltration and histological grade of infiltrating duct tumours (Table II). High grade tumours contained the most marked lymphoid reaction ($P<0.0001$). Table III summarizes the inverse association between the degree of elastosis and histological grade, elastosis being most marked in grade 1 tumours ($P=0.0001$).

*Receptor status and node metastases*

There was no association between the receptor status of the primary tumour and the number of axillary lymph nodes containing metastases ($RE_c$ *vs.* nodes, $P=0.3$; $RP_c$ *vs.* nodes, $P=0.7$). The histological findings are summarized in Table IV.

**Table II** Frequency of concurrence of each degree of lymphocytic infiltration and histological grade in 183 infiltrating duct tumours

| | *Lymphocytic infiltration* | | |
|---|---|---|---|
| | *None* | *Moderate* | *Marked* |
| Grade I ($n=49$) | 34 | 14 | 1 |
| Grade II ($n=94$) | 52 | 29 | 13 |
| Grade III ($n=40$) | 4 | 20 | 16 |

($P=<0.0001$).

**Table III** Frequency of concurrence of each degree of elastosis and histological grade in 172 infiltrating duct tumours

| | *Elastosis* | | |
|---|---|---|---|
| | *None* | *Present* | *Marked* |
| Grade I ($n=48$) | 14 | 12 | 22 |
| Grade II ($n=85$) | 30 | 35 | 20 |
| Grade III ($n=39$) | 25 | 12 | 2 |

($P=0.0001$).

*Receptors and recurrence rates*

When the follow-up data were analysed neither age nor menopausal status at the time of first presentation had any influence on the recurrence

**Figure 4** Relationship of $RE_c$ and $RP_c$ to degree of lymphocytic infiltration of primary breast cancers.

**Table IV** Summary of relationship of $RE_c$ and $RP_c$ receptor status to histological observations

| *Histological observation* | *Association with oestrogen receptor ($RE_c$)* | *Association with Progesterone receptor ($RP_c$)* |
|---|---|---|
| Diameter of primary tumour | None | None |
| Margin of primary tumour | None | None |
| Histological type | Statistically none, but all tubular and most lobular tumours $RE_c$ +ve | Statistically none, but all tubular tumours $RP_c$ +ve |
| Grade of infiltrating duct tumours | $RE_c$ occur most frequently in grade I (well-differentiated) tumours ($P=0.0004$) | $RP_c$ occur most frequently in grade I (well-differentiated) tumours ($P=0.0001$) |
| Lymphocytic infiltration | Inverse association between lymphoid reaction and presence of $RE_c$ ($P=0.0001$) | Inverse association between lymphoid reaction and presence of $RP_c$ ($P=0.0004$) |
| Epithelial cellularity: | In $RE_c$+ve tumours, highest values in most cellular tumours (post-menopausal) ($P<0.001$) but unrelated to receptor status | none |
| Stromal fibrosis | In $RE_c$+ve tumours, lowest values in those tumours with marked fibrosis (postmenopausal) ($P<0.0001$) but unrelated to receptor status. | None |
| Elastosis | $RE_c$ occur most frequently in tumours with elastosis ($P=0.02$) | $RP_c$ occur most frequently in tumours with elastosis ($P=0.004$) |
| Number of axillary nodes containing tumour | None | None |

rate of the tumour. The presence or absence or $RE_c$ and $RP_c$ was analysed with respect to the RFS 15 and 29 months after the last patient entered the study. At a median observation period of 24 months, a statistically significant increase in RFS was found for patients who possessed $RE_c$ compared with those who lacked this receptor ($P=0.02$). This difference was no longer significant at a median period of observation of 34 months (Figure 5). When the patients were divided into 4 groups according to the $RE_c$ content of the tumour ($RE_c$ negative, 5–30, 31–100, $>100\,\mathrm{fM\,mg^{-1}}$ cytosol protein) there was no statistical relationship between RFS and the $RE_c$ value at any time (Figure 6). In 163 patients $RP_c$ was also measured. The presence of this receptor was not related to RFS (Figure 7). This was noted in both $RE_c$ positive and $RE_c$ negative tumours, although the number of patients who possessed $RP_c$ and lacked $RE_c$ was very small.

The importance of the presence of metastases in the ipsilateral axillary nodes in determining the prognosis in breast cancer has been emphasised (Say & Donegan, 1974; Fisher *et al.*, 1975). This was confirmed in the present study. The overall recurrence rate was significantly higher in node +ve patients regardless of their receptor status ($P<0.001$). Three years after mastectomy 80% of patients without axillary node involvement remained free from recurrence compared with 56% of those who had 1–3 involved nodes and less than 30% of patients with 4 or more nodes containing tumour.

RFS was analysed in relation to receptor status and axillary node involvement (Figure 8). There was no statistically significant difference in the RFS between $RE_c$ +ve and $RE_c$ −ve tumours in patients without axillary metastases ($P>0.9$) or in patients with extensive nodal disease ($\geq 4$) ($P=0.2$). In patients with 1–3 axillary nodes involved, $RE_c$ +ve tumours were associated with a longer RFS than $RE_c$ −ve tumours at a median observation period of 24 months ($P=0.05$), but not at 34 months ($P=0.07$). There were no differences between the RFS of $RP_c$ +ve and $RP_c$ −ve tumours in any axillary node subgroup.

### Discussion

Breast cancer varies in its responsiveness to hormones, and is the first disease in which estimation of tissue receptors at the time of initial surgery has been advocated as a method of identifying patients with the greatest risk of recurrence. The purpose of this study was to examine inter-relationships between steroid receptor activity and a variety of histological features, and to

**Figure 5** Effect of $RE_c$ status on relapse-free survival.

assess the value of $RE_c$ and $RP_c$ as independent factors in the prognosis of breast cancer.

We have shown $RE_c$ and $RP_c$ to be independent of certain factors known to influence the natural history of the disease, for example tumour size, and the nature of its margin and axillary metastases (Fisher *et al.*, 1975). Although we could not show a statistical relationship between receptors and the histological type it is interesting to note that all tubular carcinomas had both $RE_c$ and $RP_c$. This confirms the observation of Antoniades & Spector (1979). We also confirmed reports of a high incidence of $RE_c$ in invasive lobular tumours (Rosen *et al.*, 1975; Antoniades & Spector, 1979; Martin *et al.*, 1979*b*; Rasmussen *et al.*, 1981). Other workers have failed to demonstrate any such association (Johansson *et al.*, 1970; Feherty *et al.*, 1971; Wittliff *et al.*, 1971; LeClercq *et al.*, 1973; Aspegren & Hakansson, 1974), but in most series the majority of tumours were infiltrating duct carcinomas and, as in the present study, the number of uncommon histological types was too small to permit statistical evaluation. The finding that both invasive lobular and tubular carcinomas have a high incidence of $RE_c$ supports the hypothesis that they are extreme variants of the same histological entity (Eusebi *et al.*, 1979).

Although in the past it was generally accepted that there was no association between the presence of $RE_c$ and the histological features of infiltrating duct tumours (Johansson *et al.*, 1970; Feherty *et al.*, 1971; Rosen *et al.*, 1975), only Feherty used the W.H.O. recommended system of grading. More recently, using this method exclusively, Maynard *et al.* (1978), Martin *et al.* (1979*b*), Rasmussen *et al.* (1981) and Thoresen *et al.* (1981) have shown a definite relationship between histological grade and $RE_c$.

Our data agree with the latter reports and in addition have confirmed the findings of Martin *et al.* (1979*b*) that the possession of $RP_c$ is also linked to histological grade. Terenius *et al.* (1974) was unable to show a relationship between the presence or absence of $RE_c$ and grade, but found that $RE_c$ values were statistically higher in well-differentiated tumours. The method of grading was not specified. In the present study, as in that of Heusen *et al.* (1975) and Thoresen *et al.* (1981), the mean value of the receptors was similar in all three grades, and thus the significance of the association between grade and presence or absence of receptor is difficult to interpret. Silvestrini *et al.* (1979) using thymidine-labelling indices have shown that tumours with rapid rates of cell division have a low

**Figure 6** Effect of $RE_c$ value on relapse-free survival.

incidence of $RE_c$. This, and the frequency with which receptors are found in low grade tumours suggests that receptors are associated in some way with low rates of cellular replication.

Cellularity, when assessed both at the centre and the edge of the tumour to avoid sampling errors, was unrelated to the presence of $RE_c$ and $RP_c$. However, in receptor-positive tumours, there was a significant association between the highest values of $RE_c$ and those tumours with the most abundant epithelial component. This was found both in the overall group and post-menopausal patients but not in pre-menopausal patients. Conversely, those tumours with the most marked stromal reaction had the lowest $RE_c$ values. Once again this was not seen in the pre-menopausal patients. By contrast,

**Figure 7** Effect of $RP_c$ status on relapse-free survival.

**Figure 8** Effect of $RE_c$ status and node involvement on relapse-free survival.

the mean value of the progesterone receptors was similar for all degrees of cellularity. These findings are identical with those of Masters *et al.* (1978) and similar, in part, to the results of Terenius *et al.* (1974), Antoniades & Spector (1979) and Martin *et al.* (1979*a*). Rosen *et al.* (1975) observed some association between cellularity and $RE_c$ value but did not consider it significant. Feherty *et al.* (1971) and Rasmussen *et al.* (1981) were unable to show this association. The failure to demonstrate such a link may be the result of unavoidable variation in methods used to assess cellularity (Antoniades & Spector, 1979).

It seems that although cellularity does not affect the frequency with which receptor-positive tumours are identified, it does influence the value of $RE_c$ when present and therefore $RE_c$ negativity, based on a low receptor value, should be considered with caution in tumours of low cellularity for it may only reflect the lack of epithelial cells (Martin *et al.*, 1979*a*). As the presence of $RP_c$ is normally closely linked with $RE_c$ (Martin *et al.*, 1979*b*) the dissociation of $RE_c$ and $RP_c$ in relation to cellularity is unexplained.

There is no readily apparent explanation for the observation that receptors occur infrequently in tumours with a prominent lymphocytic infiltration, a relationship which has been reported previously (Rosen *et al.*, 1975). It seems unlikely that lymphocytes *per se* are responsible for the absence of receptors within a tumour and this inverse association may merely reflect the observed statistically significant association between heavy lymphoid infiltration and poorly differentiated tumours. A lymphoid reaction is known to be closely related to the degree of malignancy (Fisher *et al.*, 1975).

The association of hormone receptors with the presence of elastic tissue within the tumour stroma may have a similar explanation for there was a statistical association between histological grade and elastosis, the latter being most marked in well-differentiated (grade I) tumours. Masters and his colleagues (1976, 1978) and Rasmussen *et al.* (1981) have also found an association between $RE_c$ and elastosis, a feature which occurs frequently in postmenopausal patients, and an association has been reported between $RP_c$ and elastosis (Rolland *et al.*, 1980). They could give no satisfying explanation for their results.

Shivas & Douglas (1972) have shown that patients whose tumours contain elastic tissue survive longer than those who lack it. They are also more likely to respond to endocrine therapy (Masters *et al.*, 1979). Well-differentiated (grade I) tumours have similar properties (McGuire *et al.*, 1977). These reports taken with the present data emphasise that hormone-dependent cells in breast tumours can be demonstrated both biochemically and histologically and many of the features of breast cancer are interdependent. Steroid receptors cannot be considered in isolation when planning treatment.

The relationship between receptor status and prognosis is not a simple one. Several reports have suggested that early relapse of breast cancer is clearly associated with a lack of $RE_c$ (Maynard *et al.*, 1978; Cooke *et al.*, 1979, 1980; Allegra *et al.*, 1979; Hahnel *et al.*, 1979; Forrest *et al.*, 1980; Westerberg *et al.*, 1980). Patients who have $RE_c$ are reported to live longer than those who lack it (Bishop *et al.*, 1979; Croton *et al.*, 1981). Kinne *et al.* (1981) and Samaan *et al.* (1981) found no overall difference in the RFS of patients with and without $RE_c$ but have found small significant differences in subgroups based on node or menopausal status. The data from the present study, like those of Hilf *et al.* (1980) fails to demonstrate any clear prognostic value for disease recurrence. Similarly there is conflicting evidence that the progesterone receptor is a reliable indicator of prognosis. Pichon *et al.* (1980) demonstrated an increase in RFS for patients with $RP_c$ whereas Allegra *et al.* (1979) and Kinne *et al.* (1981) as in the present study, found no association between the $RP_c$ and RFS.

The reasons for these conflicting results are not clear. Variations in the method of receptor analysis may be important and Forrest *et al.* (1980) showed that by merely moving the "cut-off point" separating receptor-rich (+ve) from receptor-poor (low +ve and −ve) tumours, the prognostic value for $RE_c$ could be eliminated. However, there is good qualitative agreement between centres using the DCC technique, including our own (King *et al.*, 1978), and the present study has employed similar criteria for receptor positivity to previous reports.

Hilf and his colleagues (1980) stressed that clinical factors may cause confusion in interpreting data relating $RE_c$ status to prognosis, and their observations may also apply to $RP_c$. In early studies numbers were small or receptor data from primary and secondary tumours were combined. In some, no allowance was made for patients receiving additional systemic or local treatment following mastectomy, in others, the status of the axillary nodes was not accurately known, staging being based on pectoral node biopsy, a technique which in our experience may give a false-negative rate of axillary node involvement as high as 20% (Howat & Harris, 1982).

The length of follow-up is short in many of the reports claiming prolonged RFS (Knight *et al.*, 1977; Maynard *et al.*, 1978; Pichon *et al.*, 1980), and actual survival (Bishop *et al.*, 1979; Croton *et al.*, 1981) for receptor +ve patients. Our experience has emphasised the importance of prolonged followup. At a median follow-up of 24 months our results were similar to those of Knight *et al.* (1977), Maynard *et al.* (1978) and Kinne *et al.* (1981). We observed an increase in RFS for $RE_c$ +ve patients with lymph node involvement, but not for any other sub-group. The data suggested that only those with minimal axillary disease (1–3 nodes) benefitted and it was thought that too few node −ve patients had recurred to show any difference attributable to the $RE_c$ status and that patients with extensive nodal involvement ($\geq 4$) had recurred too rapidly for any beneficial effect of $RE_c$ to become apparent. However, when the median follow-up reached 34 months the difference between $RE_c$ +ve and $RE_c$ −ve patients in the 1–3 node subgroup had disappeared. Hahnel and his colleagues (1979) made similar observations. An apparently advantageous effect of the $RE_c$ in node +ve patients seen in the first 2 years after mastectomy disappeared so that at 5 years they too were unable to demonstrate a significant difference between the RFS of $RE_c$ +ve and −ve patients in any subgroup. Von Maillot *et al.* (1982) obtained similar results for both $RE_c$ +ve and $RP_c$ +ve patients when they were compared with receptor-negative cases. Benson *et al.* (1982) made similar observations on survival. At 5 years there was no benefit for $RE_c$ +ve patients despite an earlier trend in their favour.

It is not surprising that the progesterone receptor did not influence the RFS in this study for its presence is closely linked to that of $RE_c$. Thus one might expect that the absence of any effect on RFS of the $RE_c$ would be reflected in the results obtained with $RP_c$.

As the data obtained in our study did not demonstrate any marked prognostic value for either $RE_c$ or $RP_c$ status and as the presence of these receptors may merely reflect more easily assessed histological features, we conclude that the measurement of these receptors is of no value in identifying those patients at greatest risk of recurrence. Other factors such as tumour size and axillary node status remain pre-eminent as reliable guides to prognosis.

The authors are grateful to Prof. R.A. Sellwood for permission to study patients in his care and Dr. H. Bush, Dr. A. Howell, Mr. R.N.L. Harland, Mr. M.J. Hudson and Mr. L.G. Skinner, for their interest and invaluable advice given during the preparation of this paper. Ms E. Hayward gave expert technical assistance and Ms L. Shaughnessy typed the manuscript.

### References

ALLEGRA, J.C., LIPPMAN, M.E., SIMON, R. & 7 others. (1979). Association between steroid hormone receptor status and disease free interval in breast cancer. *Cancer Treat. Rep.*, **63**, 1271.

ANTONIADES, K. & SPECTOR, H. (1979). Correlation of oestrogen receptor levels with histology and cytomorphology in human mammary cancer. *Am. J. Clin. Pathol.*, **71**, 497.

ASPEGREN, K. & HAKANSSON, L. (1974). Human mammary carcinoma studied for hormone responsiveness in short term incubations. *Acta. Chir. Scand.*, **140**, 95.

BARNES, D.M., RIBEIRO, G.G. & SKINNER, L.G. (1977). Two methods for measurement of oestradiol-17$\beta$ and progesterone receptors in human breast cancer and correlation with response to treatment. *Eur. J. Cancer*, **13**, 1133.

BENSON, E.A., CARTWRIGHT, R.A., COWEN, P.N. & HAMILTON, J. (1982). Oestrogen receptors and survival in early breast cancer. *Br. Med. J.*, **284**, 597.

BISHOP, H.M., BLAMEY, R.W., ELSTON, C.W., HAYBRITTLE, J.L., NICHOLSON, R.I. & GRIFFITHS, K. (1979). Relationship of oestrogen-receptor status to survival in breast cancer. *Lancet*, **ii**, 283.

BLOOM, H.J.G. & RICHARDSON, W.W. (1957). Histological grading and prognosis in breast cancer. *Br. J. Cancer*, **11**, 359.

COOKE, T., GEORGE, W.D. & GRIFFITHS, K. (1980). Possible tests for selection of adjuvant systemic therapy in early cancer of the breast. *Br. J. Surg.*, **67**, 747.

COOKE, T., GEORGE, D., SHIELDS, R., MAYNARD, P. & GRIFFITHS, K. (1979). Oestrogen receptors and prognosis in breast cancer. *Lancet*, **i**, 995.

CROTON, R., COOKE, T., HOLT, S., GEORGE, W.D., NICHOLSON, R. & GRIFFITHS, K. (1981). Oestrogen receptors and survival in early breast cancer. *Br. Med. J.*, **283**, 1289.

EUSEBI, V., BETTS, C.M. & BUSSOLATI, G. (1979). Tubular carcinoma: a variant of secretory breast carcinoma. *Histopathology*, **3**, 407.

FEHERTY, P., FARRER-BROWN, G. & KELLIE, A.E. (1971). Oestradiol receptors in carcinoma and benign disease of the breast: An *in vitro* assay. *Br. J. Cancer*, **25**, 697.

FISHER, E.R., GREGORIO, R.M., FISHER, B., REDMOND, C., VELLIOS, F. & SOMMERS, S.C. (1975). The pathology of invasive breast cancer. *Cancer*, **36**, 1.

FORREST, A.P.M., BLACK, R.B., HUMENIUK, V. & 8 others (1980). Preoperative assessment and staging of breast cancer: preliminary communication. *J. R. Soc. Med.*, **73**, 561.

HAHNEL, R., WOODINGS, T. & VIVIAN, A.B. (1979). Prognostic value of oestrogen receptors in primary breast cancer. *Cancer*, **44**, 671.

HEUSEN, J.C., LECLERCQ, G., LONGEVAL, E., DEBOEL, M.C., MATTHEIEM, W.H. & HEIMANN, R. (1975). Estrogen receptors: Prognostic significance in breast cancer. In *Estrogen Receptors in Human Breast Cancer*, (Eds. McGuire *et al.*) New York: Raven Press. p. 57.

HILF, R., FELDSTEIN, M.L., SCOTT, G.L. & SAVLOV, E.D. (1980). The relative importance of estrogen receptor analysis as a prognostic factor for recurrence or response to chemotherapy in women with breast cancer. *Cancer*, **45**, 1993.

HOWAT, J.M.T. & HARRIS, M. (1982). Axillary sampling in breast cancer. *Lancet*, **ii**, 37.

JOHANSSON, H., TERENIUS, L. & THOREN, L. (1970). The binding of estradiol-17$\beta$ to human breast cancer and other tissues *in vitro*. *Cancer Res.*, **30**, 692.

KING, R.J.B., BARNES, D.M., HAWKINS, R.A., LEAKE, R.E., MAYNARD, P.V. & ROBERTS, M.M. (1978). Measurement of oestradiol receptors by five institutions on common tissue samples. *Br. J. Cancer*, **38**, 428.

KINNE, D.W., ASHIKARI, R., BUTLER, A., MENENDEZ-BOTET, C., ROSEN, P.P. & SCHWARTZ, M. (1981). Estrogen receptor protein in breast cancer as a predictor of recurrence. *Cancer*, **47**, 2364.

KNIGHT, W.A., LIVINGSTON, R.B., GREGORY, E.J. & MCGUIRE, W.L. (1977). Estrogen receptor as an independent prognostic factor for early recurrence in breast cancer. *Cancer Res.*, **37**, 4669.

KORENMAN, S.G. & DUKES, B.A. (1970). Specific oestrogen binding by the cytoplasm of human breast carcinoma. *J. Clin. Endocrinol.*, **30**, 639.

LECLERCQ, G., HEUSON, J.C., SCHOENFIELD, R., MATTHEIEM, W.J. & TAGNON, H.J. (1973). Oestrogen receptors in human breast cancer. *Eur. J. Cancer*, **9**, 665.

MARTIN, P.M., ROLLAND, P.H., JACQUEMIER, J., ROLLAND, A.M. & TOGA, M. (1979*a*). Multiple steroid receptors in human breast cancer. II Oestrogen and progestin receptors in 672 primary tumours. *Cancer Chemother. Pharmacol.*, **2**, 107.

MARTIN, P.M., ROLLAND, P.H., JACQUEMIER, J., ROLLAND, A.M. & TOGA, M. (1979*b*). Multiple steroid receptors in human breast cancer. III Relationships between steroid receptors and the state of differentiation and the activity of carcinomas throughout the pathological features. *Cancer Chemother. Pharmacol.*, **2**, 115.

MASTERS, J.R.W., HAWKINS, R.A., SANGSTER, K. & 5 others (1978). Oestrogen receptors, cellularity, elastosis and menstrual status in human breast cancer. *Eur. J. Cancer*, **14**, 303.

MASTERS, J.R.W., MILLIS, R.R., KING, R.J.B. & RUBENS, R.D. (1979). Elastosis and response to endocrine therapy in human breast cancer. *Br. J. Cancer*, **39**, 536.

MASTERS, J.R.W., SANGSTER, K., HAWKINS, R.A. & SHIVAS, A.A. (1976). Elastosis and oestrogen receptors in human breast cancer. *Br. J. Cancer*, **33**, 342.

MAYNARD, P.V., BLAMEY, R.W., ELSTON, C.W., HAYBRITTLE, J.L. & GRIFFITHS, K. (1978). Estrogen receptor assay in primary breast cancer and early recurrence of disease. *Cancer Res.*, **38**, 4292.

MAYNARD, P.V., DAVIES, C.J., BLAMEY, R.W., ELSTON, C.W., JOHNSTON, J. & GRIFFITHS, K. (1978). Relationship between oestrogen receptor content and histological grade in human primary breast tumour. *Br. J. Cancer*, **38**, 745.

MCGUIRE, W.L., HORWITZ, K.B., PEARSON, O.H. & SEGALOFF, A. (1977). Current status of oestrogen and progesterone receptors in breast cancer. *Cancer*, **39**, 2934.

MILLIS, R.R., RUBENS, R.D., MASTERS, J.R.W. & MINTON, M.J. (1981). Histological grade and response to endocrine therapy in breast cancer. *Lancet*, **ii**, 101.

PETO, R., PIKE, M.C., ARMITAGE, P. & 7 others. (1977). Design and analysis of randomised clinical trials requiring prolonged observation of each patient. II Analysis and examples. *Br. J. Cancer*, **35**, 1.

PICHON, M.G., PALLUD, C., BRUNET, M. & MILGROM, E. (1980). Relationship of presence of progesterone receptors to prognosis in early breast cancer. *Cancer Res.*, **40**, 3357.

RASMUSSEN, B.B., ROSE, C., THORPE, S.M., HOU-JENSEN, K., DAEHNFELDT, J.L. & PALSHOF, T. (1981). Histopathological characteristics and oestrogen receptor content in primary breast cancer. *Vichows Arch.* (*Pathol. Anat.*), **390**, 347.

ROLLAND, P.H., JACQUEMIER, J. & MARTIN, P.M. (1980). Histological differentiation in human breast cancer is related to steroid receptors and stromal elastosis. *Cancer Chemother. Pharmacol.*, **5**, 73.

ROSEN, P.P., MENENDEZ-BOTET, C.J., NISSELBAUM, J.S. & 4 others. (1975). Pathological review of breast lesions analysed for estrogen receptor protein. *Cancer Res.*, **35**, 3187.

SANDER, S. (1968). The *in vitro* uptake of oestradiol in biopsies from 25 breast cancer patients. *Acta. Pathol. Microbiol. Scand.*, **74**, 301.

SAMAAN, N.A., BUZDAR, A.U., ALDINGER, K.A. & 4 others (1981). Estrogen receptor: a prognostic factor in breast cancer. *Cancer*, **47**, 554.

SAY, C.C. & DONEGAN, W.L. (1974). Invasive carcinoma of the breast: Prognostic significance of tumour size and involved axillary lymph nodes. *Cancer*, **34**, 468.

SCARFF, R.W. & TORLONI, H. (1968). Histological typing of breast tumours. In: *International Histological Classification of Tumours* No. 2, p. 17, Geneva: WHO.

SCATCHARD, G. (1949). The attraction of proteins for small molecules and ions. *Ann. N.Y. Acad. of Science*, **51**, 660.

SHIVAS, A.A. & DOUGLAS, J.G. (1972). The prognostic significance of elastosis in breast cancer. *J. R. Col. Surg.* (Edin.) **17**, 315.

SILVESTRINI, R., DAIDONE, M.G. & DIFRONZO, G. (1979). Relationship between proliferative activity and estrogen receptors in breast cancer. *Cancer*, **44**, 665.

SKINNER, L.G., BARNES, D.M. & RIBEIRO, G.G. (1980). The clinical value of multiple steroid receptor assay in breast cancer management. *Cancer*, **46**, 2939.

TERENIUS, L., JOHANSSON, H., RIMSTEN, Å. & THORÉN, L. (1974). Malignant and benign mammary disease: estrogen binding in relation to clinical data. *Cancer*, **33**, 1364.

THORESEN, S., TANGEN, M., STØA, K.F. & HARTVEIT, F. (1981). Oestrogen receptor values and histological grade in breast cancer. *Histopathology*, **5**, 257.

VON MAILLOT, K., HORKE, W. & PRESTELE, H. (1982). Prognostic significance of the steroid receptor content in primary breast cancer. *Arch. Gynaecol.*, **231**, 185.

WESTERBERG, H., GUSTAFSON, S.A., NORDENSKJOLD, B., SILEVERSWARD, C. & WALLGREN, A. (1980). Estrogen receptor level and other factors in early recurrence of breast cancer. *Int. J. Cancer*, **26**, 429,

WITTLIFF, V.L., HILF, R., BROOKS, W.F., SAVLOV, E.D., HALL, T.C. & ORLANDO, R.A. (1971). Specific estrogen binding capacity of the cytoplasmic receptor in normal and neoplastic breast tissues in humans. *Cancer Res.*, **32**, 1983.